# Nitroalkene fatty acids modulate bile acid metabolism and lung function in obese asthma

**DOI:** 10.1038/s41598-021-96471-9

**Published:** 2021-09-07

**Authors:** Michelle L. Manni, Victoria A. Heinrich, Gregory J. Buchan, James P. O’Brien, Crystal Uvalle, Veronika Cechova, Adolf Koudelka, Dharti Ukani, Mohamad Rawas-Qalaji, Tim D. Oury, Renee Hart, Madeline Ellgass, Steven J. Mullett, Merritt L. Fajt, Sally E. Wenzel, Fernando Holguin, Bruce A. Freeman, Stacy G. Wendell

**Affiliations:** 1 Division of Pulmonary Medicine, Department of Pediatrics, UPMC Children’s Hospital of Pittsburgh, Pittsburgh, PA 15224 USA; 2 Department of Pharmacology and Chemical Biology, School of Medicine, University of Pittsburgh, 200 Lothrop Street, E1345, Pittsburgh, PA 15261 USA; 3 Department of Cell Biology and Radiobiology, Institute of Biophysics, Czech Academy of Sciences, 61265 Brno, Czech Republic; 4 Department of Pathology, School of Medicine, University of Pittsburgh, Pittsburgh, PA 15261 USA; 5 Health Sciences Metabolomics and Lipidomics Core, University of Pittsburgh, Pittsburgh, PA 15261 USA; 6 Division of Pulmonary, Allergy and Critical Care Medicine, Department of Medicine, University of Pittsburgh, Pittsburgh, PA 15261 USA; 7 Department of Environmental and Occupational Health, Graduate School of Public Health, University of Pittsburgh, Pittsburgh, PA 15261 USA; 8 Division of Pulmonary Sciences and Critical Care, School of Medicine, University of Colorado Denver, Aurora, CO 80045 USA; 9 Department of Clinical and Translational Science, University of Pittsburgh, Pittsburgh, PA 15261 USA

**Keywords:** Diseases, Metabolic disorders, Respiratory tract diseases, Medical research, Experimental models of disease

## Abstract

Bile acid profiles are altered in obese individuals with asthma. Thus, we sought to better understand how obesity-related systemic changes contribute to lung pathophysiology. We also test the therapeutic potential of nitro-oleic acid (NO_2_-OA), a regulator of metabolic and inflammatory signaling pathways, to mitigate allergen and obesity-induced lung function decline in a murine model of asthma. Bile acids were measured in the plasma of healthy subjects and individuals with asthma and serum and lung tissue of mice with and without allergic airway disease (AAD). Lung function, indices of inflammation and hepatic bile acid enzyme expression were measured in obese mice with house dust mite-induced AAD treated with vehicle or NO_2_-OA. Serum levels of glycocholic acid and glycoursodeoxycholic acid clinically correlate with body mass index and airway hyperreactivity whereas murine levels of β-muricholic acid and tauro-β-muricholic acid were significantly increased and positively correlated with impaired lung function in obese mice with AAD. NO_2_-OA reduced murine bile acid levels by modulating hepatic expression of bile acid synthesis enzymes, with a concomitant reduction in small airway resistance and tissue elastance. Bile acids correlate to body mass index and lung function decline and the signaling actions of nitroalkenes can limit AAD by modulating bile acid metabolism, revealing a potential pharmacologic approach to improving the current standard of care.

## Introduction

Obesity induces a chronic systemic inflammatory state characterized by impaired adipokine signaling, pro-inflammatory cytokine production, immune cell activation and enhanced generation of oxygen and nitrogen oxide-derived reactive species. Obesity is a risk factor for the development of asthma and is associated with worsening symptoms and poor asthma control^1,2^. Obese individuals with asthma often have severe, refractory disease with higher rates of exacerbations and hospitalizations^1–3^. Currently, 60% of severe asthmatic adults are obese^4^. The relationship between obesity and asthma derives from multiple physiological, environmental, and clinical factors, with specific pathogenic mechanisms remaining to be defined.

Bile acids are sterol metabolites produced by a combination of hepatic and microbial metabolic reactions and are implicated in obesity-associated changes in lung function and a pro-asthma phenotype^5,6^. Bile acids bind nuclear receptors, including the farnesoid X receptor (FXR), pregnane X receptor, vitamin D receptor, and G protein-coupled receptors like G-protein coupled bile acid receptor 1 and sphingosine 1 phosphate receptor to regulate gut barrier integrity, metabolism and their own synthesis^7,8^. Depending on their structure and the receptor identity, bile acids can act as agonists or antagonists^9–11^. Similar to short chain fatty acids, obesity-induced gut dysbiosis alters bile acid profiles and signaling^12–14^. Metabolomics studies have identified changes in bile acid profiles in individuals with asthma or food allergies; however, the relationship between altered bile acid profiles and lung function remains unclear^6,15,16^.

To elucidate the link between bile acids and lung function, we examined plasma bile acids using stable isotope dilution liquid chromatography high resolution mass spectrometry (SID-LC-HRMS). We found that bile acids are altered by obesity and asthma status, with specific bile acid levels correlating with percent predicted forced expiratory volume (FEV_1_) in adults with and without asthma. Using a murine model of obese allergic airway disease (AAD), we reveal that bile acid levels are strongly linked with lung function parameters. Specifically, pulmonary sensitization and challenge with house dust mite (HDM) allergen enhances bile acid synthesis in the liver of obese mice adversely affecting lung function. As there are few phenotype-specific treatments for obesity-associated asthma, we hypothesized that the small molecule electrophile, nitro-oleic acid (NO_2_-OA) would mitigate airway hyperreactivity. NO_2_-OA reduces metabolic syndrome, hepatic dysfunction and pulmonary hypertension in diet-induced murine models of obesity^17–19^. Thus, NO_2_-OA may modulate key gene expression and intermediary metabolism pathways that contribute to the obesity-associated asthma phenotype.

## Results

### Individuals with increased body mass index (BMI) and asthma have higher systemic levels of bile acids that correlate with decreased FEV_1_

**Figure 1:**
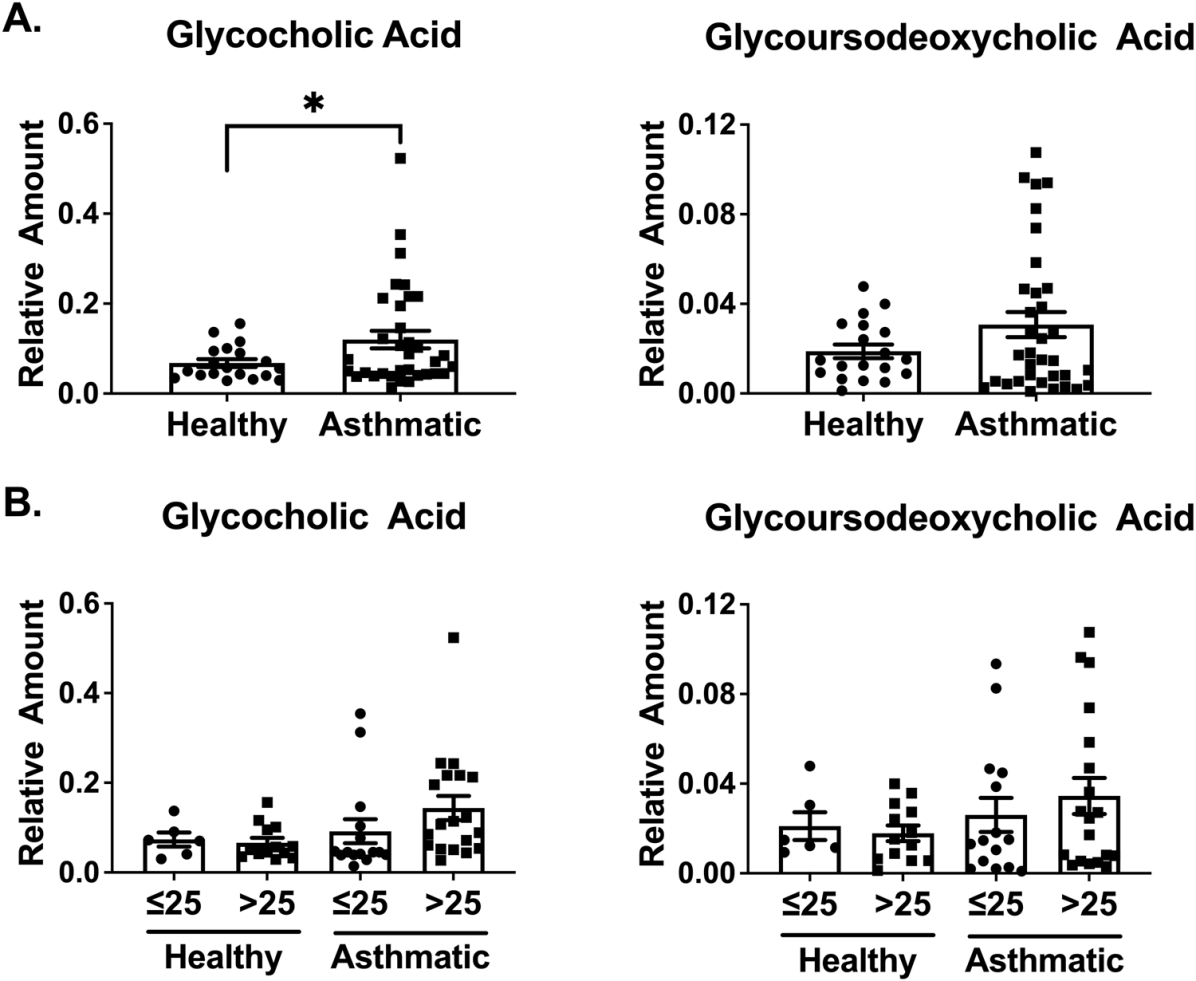
Human plasma bile acid profiles are altered with obesity and asthma. Glycocholic acid (GCA) and glycoursodeoxycholic acid (GUDCA) levels were measured by stable isotope dilution-liquid chromatography-high resolution mass spectrometry (SID-LC-HRMS) in the plasma of individuals with asthma (n = 34) and controls (n = 19) **(A)**. GCA and GUDCA levels are also shown for healthy and asthmatic patients grouped by BMI: individuals with asthma with a BMI > 25 (n = 19), lean healthy controls with a BMI > 25 (n = 6), healthy individuals with a BMI > 25 (n = 13), and lean individuals with asthma (n = 15) **(B)**. *p < 0.05.

Plasma samples from two cohorts of lean and obese healthy individuals and individuals with asthma (Table 1) were analyzed for a panel of 17 bile acids using SID-LC-HRMS. Plasma levels of glycocholic acid (GCA) were significantly increased in individuals with asthma compared to healthy subjects (p = 0.018) and glycoursodeoxycholic acid (GUDCA) trended towards significance (p = 0.066) (Fig. 1A). When GCA and GUDCA levels were stratified by BMI, the highest concentrations were detected in individuals with asthma and a BMI > 25 (Fig. 1B).

**Table 1:** Subject characteristics.

	Healthy		Asthma		p-value
	BMI ≤ 25	BMI > 25	BMI ≤ 25	BMI > 25	
	n = 6	n = 13	n = 15	n = 19	
Age (year)*	21.5 (18.8–28)	29 (24.5–36)	27 (20–32)	32 (24–51)	0.10
Sex, % female	83%	85%	87%	68%	0.54
Race (White/Black/other)	4/1/1	13/0/0	13/2	9/9/0**	0.003
BMI*	22.5 (21.1–23.9)	31.9 (28.4–39.2)	21 (20.2–23.1)	30.5 (28–35.7)	< 0.0001​
FeNO (ppb)*	16 (7.5–25)	12 (9.5–16.5)	20 (9–38)	15 (12–37)	0.26
FEV1%p*	96.5 (87.3–106.3)	101 (87–106)	95 (82–102)	89 (75–108)	0.63
FEV1/FVC*	96.5 (93.8–101)	93 (85–104)	91 (80–95)	83 (77–94)	0.08
ICS use, %	n/a	n/a	33%	39%	
ICS/LABA use, %	n/a	n/a	20%	28%	
SABA use, %	n/a	n/a	100%	100%	

*BMI* body mass index, *FEV1* forced expiratory volume in 1 s, *FVC* forced vital capacity, *FeNO* fractional exhaled nitric oxide, *ICS *inhaled corticosteroid, *LABA* long-acting beta-agonist, *SABA* short-acting beta-agonist.

*Data presented as median 25th–75th interquartile range using Wilcoxon/ANOVA. For categorical variables, Pearson X^2^ was used.

**One subject did not report race.

**Figure 2:**
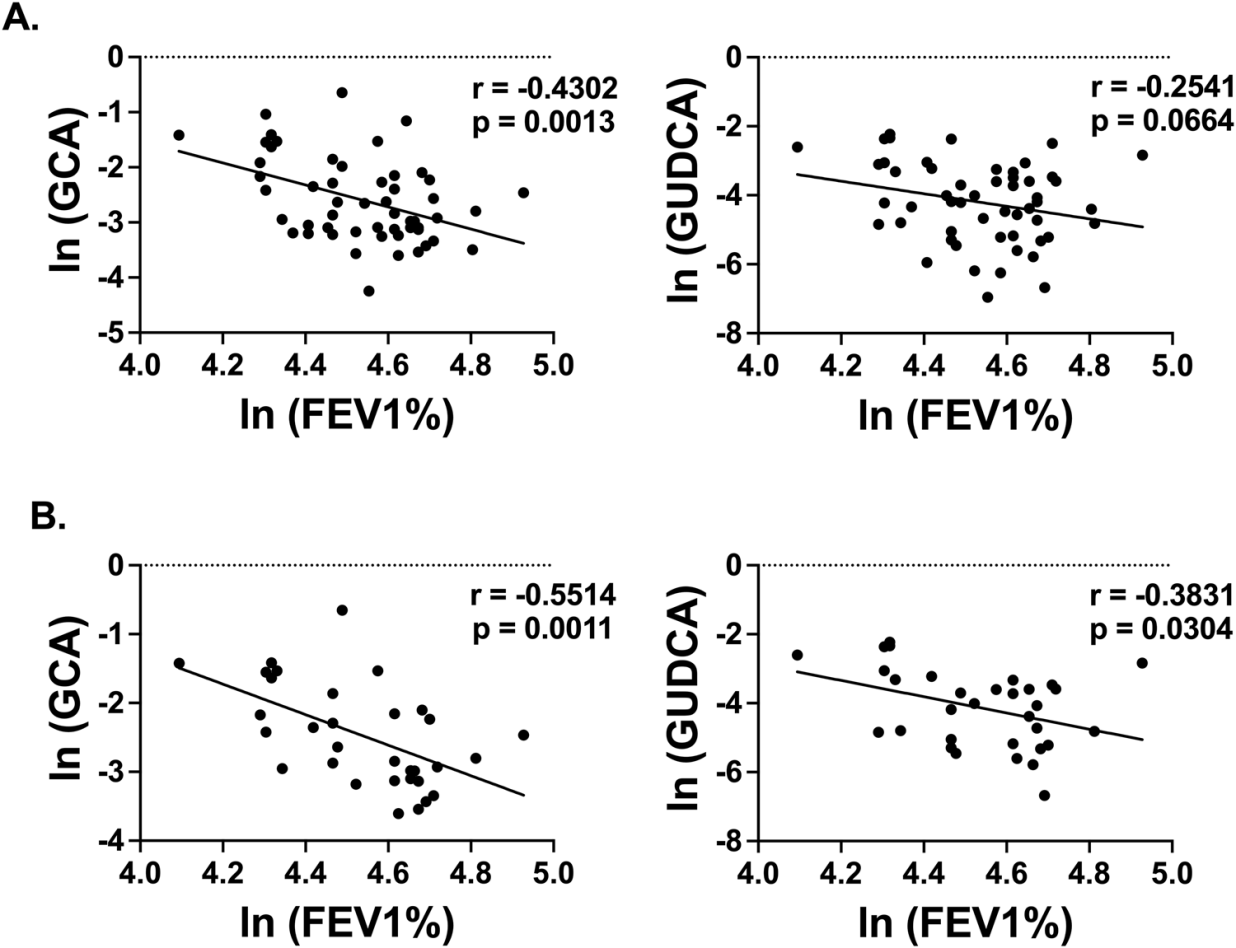
GCA and GUDCA correlate to a decrease in lung function in asthmatic individuals with high BMI. Pearson correlation of plasma glycocholic acid (GCA) and glycoursodeoxycholic acid (GUDCA) levels with FEV_1_ for all individuals (**A**, n = 53 pairs) and for those with a BMI > 25 (**B**, n = 32 pairs). Graphs show natural log transformed data.

While neither GUDCA or GCA significantly correlated with fractional exhaled nitric oxide or bronchoalveolar lavage (BAL) eosinophils, there was a trend towards a negative correlation with GUDCA and blood eosinophil levels in the Pittsburgh cohort (Fig. S1). However, for all subjects, plasma levels of GCA significantly correlated with FEV_1_ and GUDCA trended towards a significant correlation (Fig. 2A). Furthermore, analysis of only healthy and asthmatic individuals with a BMI > 25, resulted in a stronger negative correlation with FEV_1_ for GUDCA whereas the GCA correlation to BMI > 25 was similar to the correlation of FEV_1_ for all subjects, indicating that BMI may independently affect the levels of some bile acids more than others (Fig. 2B).

### NO_2_-OA improves lung mechanics in obese mice with AAD but does not diminish inflammation

To model obesity-associated asthma, C57BL/6J mice were fed 60% high fat diet chow for 12 weeks before sensitization and challenge with HDM to induce AAD. The average weights of each group of obese mice were similar prior to the induction of AAD and at the time of sacrifice (Fig. S2). NO_2_-OA (equimolar 9-NO_2_- and 10-NO_2_-oleic acid regioisomers) activates the expression of multiple tissue defense mechanisms and inhibits pro-inflammatory signaling responses in vitro and in vivo^17,18,20^. To investigate the therapeutic potential of NO_2_-OA, obese mice with AAD were gavaged with either triolein (vehicle) or NO_2_-OA.

Markers of HDM-induced inflammation were examined in our model of obese asthma. While HDM sensitization and challenge resulted in an overall increase in airway inflammation compared to obese controls (Fig. S3), NO_2_-OA treatment did not mitigate the overall inflammatory response except for a significant decrease in total BAL fluid cell counts; although, no one specific immune cell subset was significantly reduced (Fig. S3A,B). Histological assessment of pulmonary inflammation revealed an increase in inflammation in obese mice with AAD when compared to obese control mice without AAD; however, this inflammation was not significantly decreased with NO_2_-OA administration (Figs. S3C and S3D). Lastly, pro-inflammatory Th2- and Th17-related cytokines and chemokines are elevated (Figs. S3E and S3F), while the anti-inflammatory cytokine, IL-10, is decreased in the lungs of obese mice with AAD compared to obese control mice. Inflammatory mediator expression was not diminished with NO_2_-OA administration (Fig. S3G).

**Figure 3:**
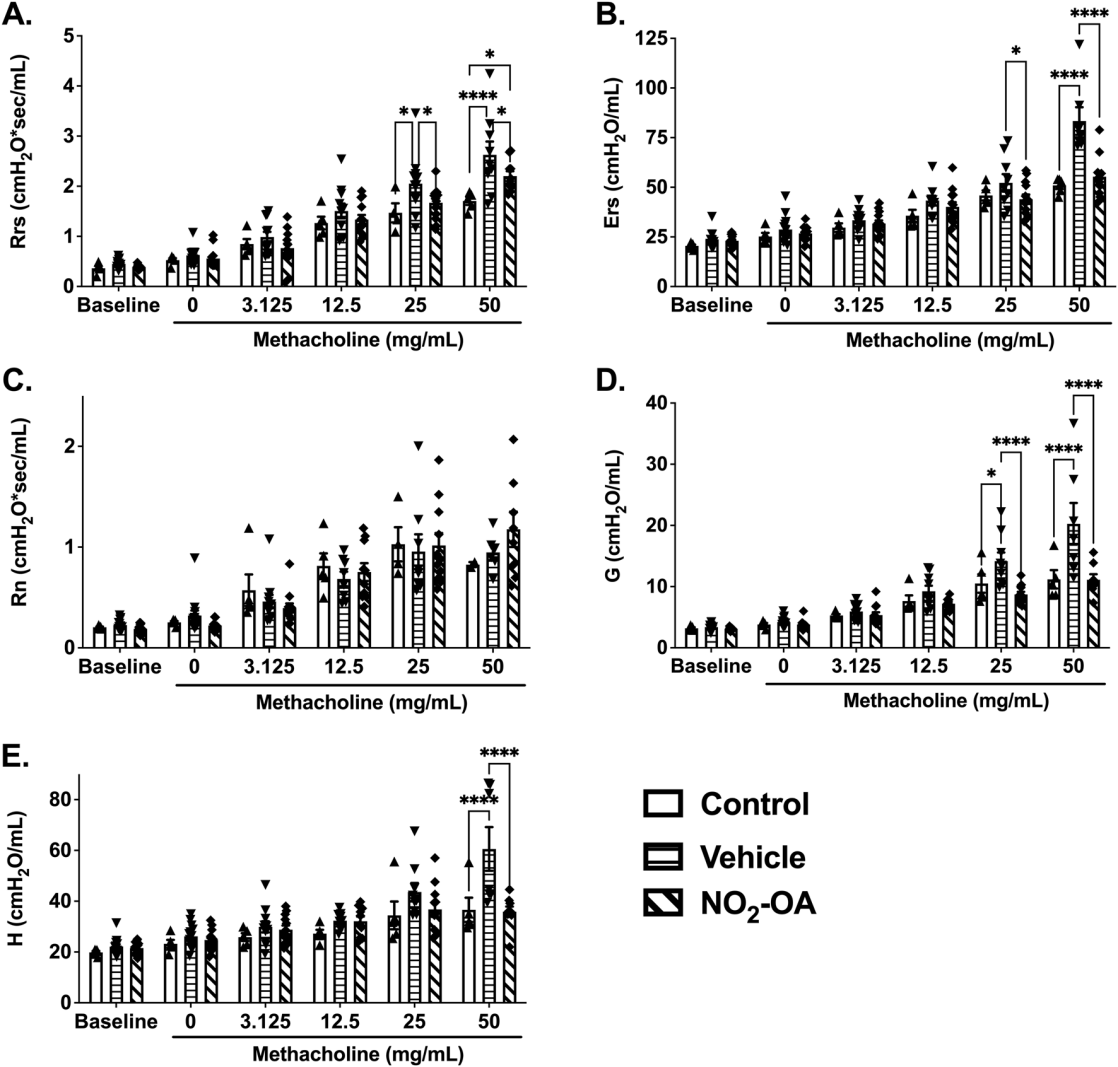
NO_*2*_-OA improves lung function in obese mice with AAD. Lung function was evaluated following NO_2_-OA treatment in obese mice with HDM-induced allergic airway disease. **(A)** Pulmonary resistance (Rrs), **(B)** pulmonary elastance (Ers), **(C)** airway resistance (Rn), **(D)** tissue damping **(G)**, and **(E)** tissue elastance **(H)** were measured at baseline and following administration of increasing doses of methacholine using a flexiVent system. Graphs show data for control (n = 2–5), vehicle (n = 6–13), and NO_2_-OA (n = 8–13) and combined from four independent experiments. *p < 0.05, ****p < 0.0001.

Lung mechanics were evaluated in obese mice with AAD following NO_2_-OA treatment. Pulmonary resistance (Rrs) and elastance (Ers) were significantly decreased in obese mice with AAD treated with NO_2_-OA compared to obese mice with AAD treated with vehicle (Fig. 3A,B). Although central airway resistance (Rn) was unaltered by NO_2_-OA treatment, tissue damping (G) observed was significantly reduced in obese mice with AAD following NO_2_-OA treatment (Fig. 3C,D). NO_2_-OA also decreased tissue elastance (H, Fig. 3E) in the lungs of obese mice with AAD at the highest dose of methacholine (50 mg/mL). Overall, these results affirm that airway hyperresponsiveness in obesity-associated AAD was attenuated by NO_2_-OA, illustrating a therapeutic potential in this subset of disease.

### Bile acid profiles are altered in AAD and correlate with lung function

**Figure 4:**
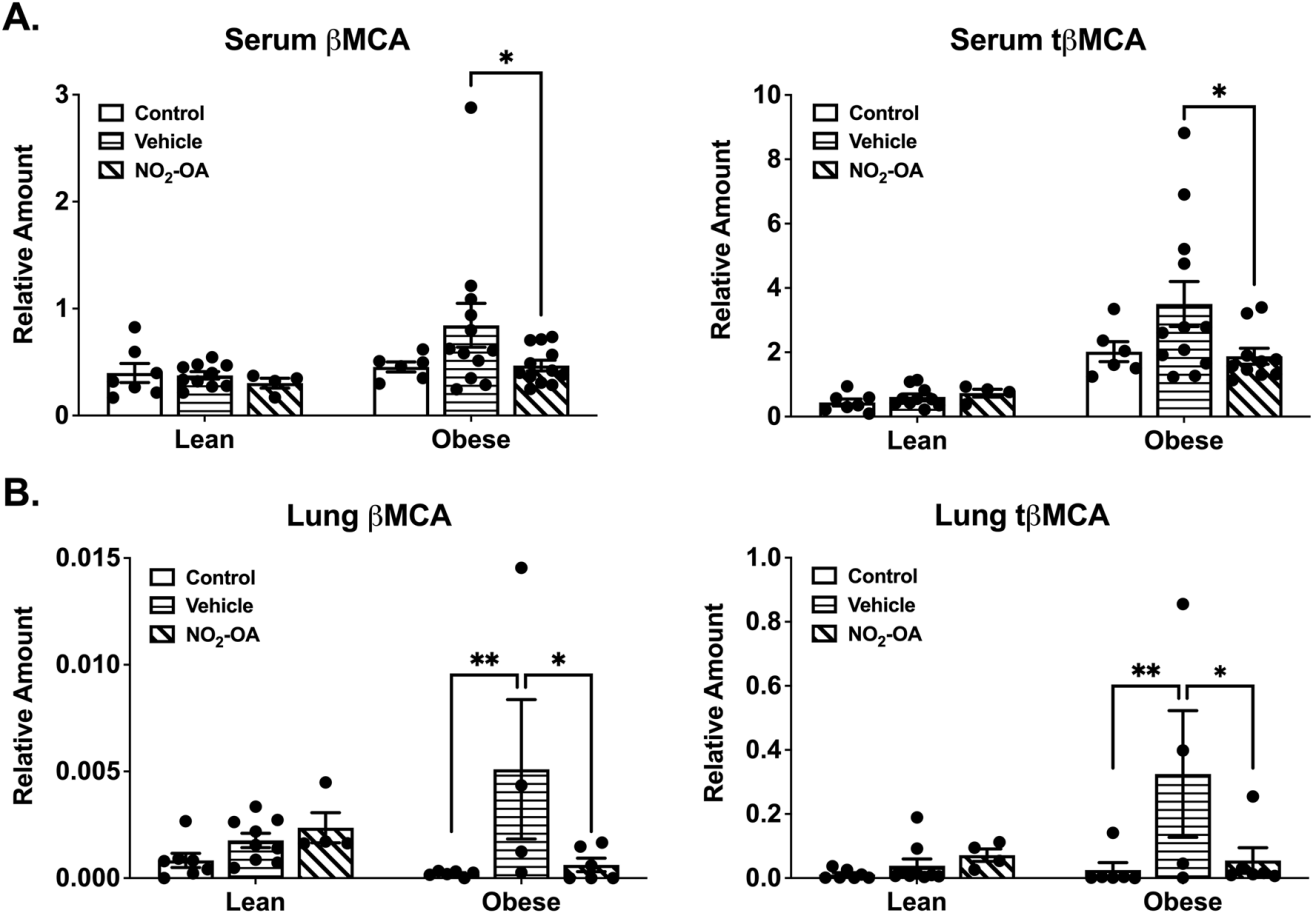
Murine bile acids increase in obese mice with AAD, and NO_2_-OA treatment reduces endogenous bile acids in these mice. β-muricholic (βMCA) and tauro-β-muricholic acid (tβMCA) levels were measured in serum **(A)** and lung **(B)** from lean and obese mice with and without AAD and NO_2_-OA treatment using SID-LC-HRMS. Graphs show data for n = 4–12 per group and combined from four independent experiments. *p < 0.05, **p < 0.01.

SID-LC-HRMS analysis of bile acids (Table 2) was performed on the serum and lung homogenates of lean and obese control mice, and lean and obese mice with AAD following NO_2_-OA or vehicle treatment. β-muricholic acid (βMCA) and tauro-β-muricholic acid (tβMCA) were increased in the serum (Fig. 4A) and lungs (Fig. 4B) of obese mice with AAD compared to obese controls and all groups of lean mice. Treatment with NO_2_-OA significantly reduced βMCA and tβMCA to levels in serum comparable to obese mice without AAD. Similarly, NO_2_-OA decreased βMCA and tβMCA levels in the lungs when compared to obese mice with AAD that received vehicle (Fig. 4A,B).

**Table 2:** LC-HRMS parameters for bile acid analysis.

Bile acid	Formula	M–H^+^	Internal standard	M–H^+^	Retention time
Cholic acid	C_24_H_40_O_5_	407.2802	CA-d_4_	411.3049	7.1
β-Muricholic acid	C_24_H_40_O_5_	407.2802	bMCA-d_5_	412.3111	5.4
Deoxycholic acid	C_24_H_40_O_4_	391.2853	DCA-d_4_	395.3100	8.8
Chenodeoxycholic acid	C_24_H_40_O_4_	391.2853	CDCA-d_4_	395.3100	8.6
Ursodeoxycholic acid	C_24_H_40_O_4_	391.2853	UDCA-d_4_	395.3100	6.3
Lithocholic acid	C_24_H_40_O_3_	375.2904	LCA-d_4_	379.3151	10.0
Glycocholic acid	C_26_H_43_NO_6_	464.3017	GCA-d_4_	468.3264	6.2
Glycodeoxycholic acid	C_26_H_43_NO_5_	448.3068	GDCA-d_4_	452.3315	8.1
Glycochenodeoxycholic acid	C_26_H_43_NO_5_	448.3068	GCDCA-d_4_	452.3315	7.7
Glycoursodeoxycholic acid	C_26_H_43_NO_5_	448.3068	GUDCA-d_4_	452.3315	5.4
Glycolithocholic acid	C_26_H_43_NO_4_	432.3119	GLCA-d_4_	436.3366	9.3
Taurocholic acid	C_26_H_45_NO_7_S	514.2843	TCA-d_4_	518.3090	6.2
Tauro-β-muricholic acid	C_26_H_45_NO_7_S	514.2843	TbMCA-d_4_	518.3090	3.9
Taurodeoxycholic acid	C_26_H_45_NO_6_S	498.2894	TDCA-d_5_	503.3203	8.1
Taurochenodeoxycholic acid	C_26_H_45_NO_6_S	498.2894	TCDCA-d_4_	502.3141	7.6
Tauroursodeoxycholic acid	C_26_H_45_NO_6_S	498.2894	TUDCA-d_4_	502.3141	5.1
Taurolithocholic acid	C_26_H_45_NO_5_S	482.2945	TLCA-d_5_	487.3254	9.2

**Figure 5:**
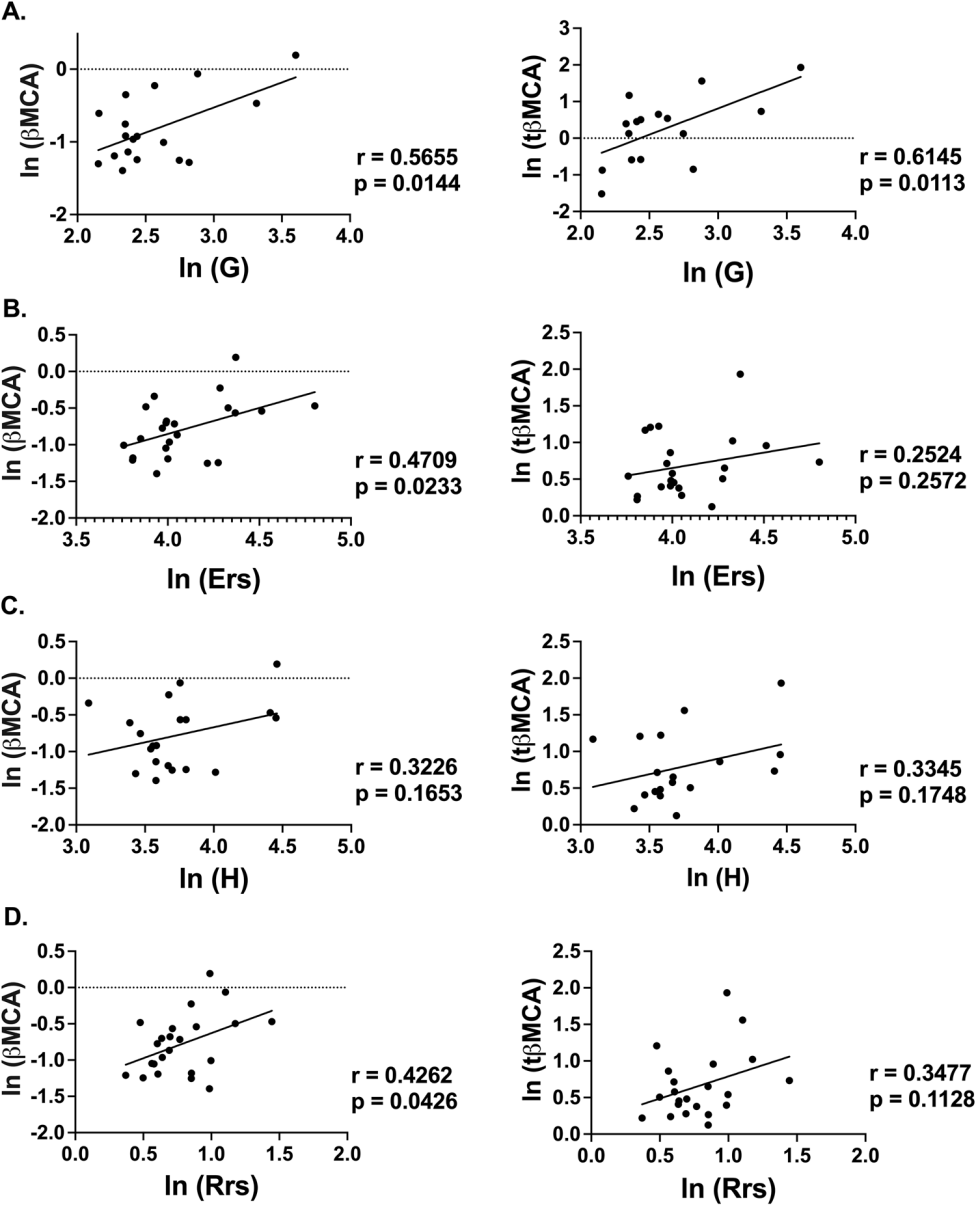
βMCA and tβ-MCA correlate with a decline of lung function in obese mice. Pearson correlations of serum βMCA and tβMCA levels with tissue damping, G **(A)**, pulmonary elastance, Ers **(B),** tissue elastance, H **(C)**, and pulmonary resistance, Rrs **(D)** measured by flexiVent following 50 mg/mL methacholine challenge in obese mice. n = 16–23 pairs. Graphs show natural log transformed data combined from four independent experiments.

Levels of βMCA and tβMCA were correlated with lung mechanics in obese mice with and without AAD and following NO_2_-OA or vehicle treatment. Both serum βMCA and tβMCA levels positively correlated with changes in small airway resistance at the highest dose of methacholine (G, Fig. 5A); however, there was no significant correlation with tissue elastance at the highest dose of methacholine (H, Fig. 5B). βMCA also positively correlated with Ers (Fig. 5C) and Rrs (Fig. 5D) at highest dose of methacholine.

### Bile acid synthesis in the liver altered during AAD is abrogated by NO_2_-OA treatment

**Figure 6:**
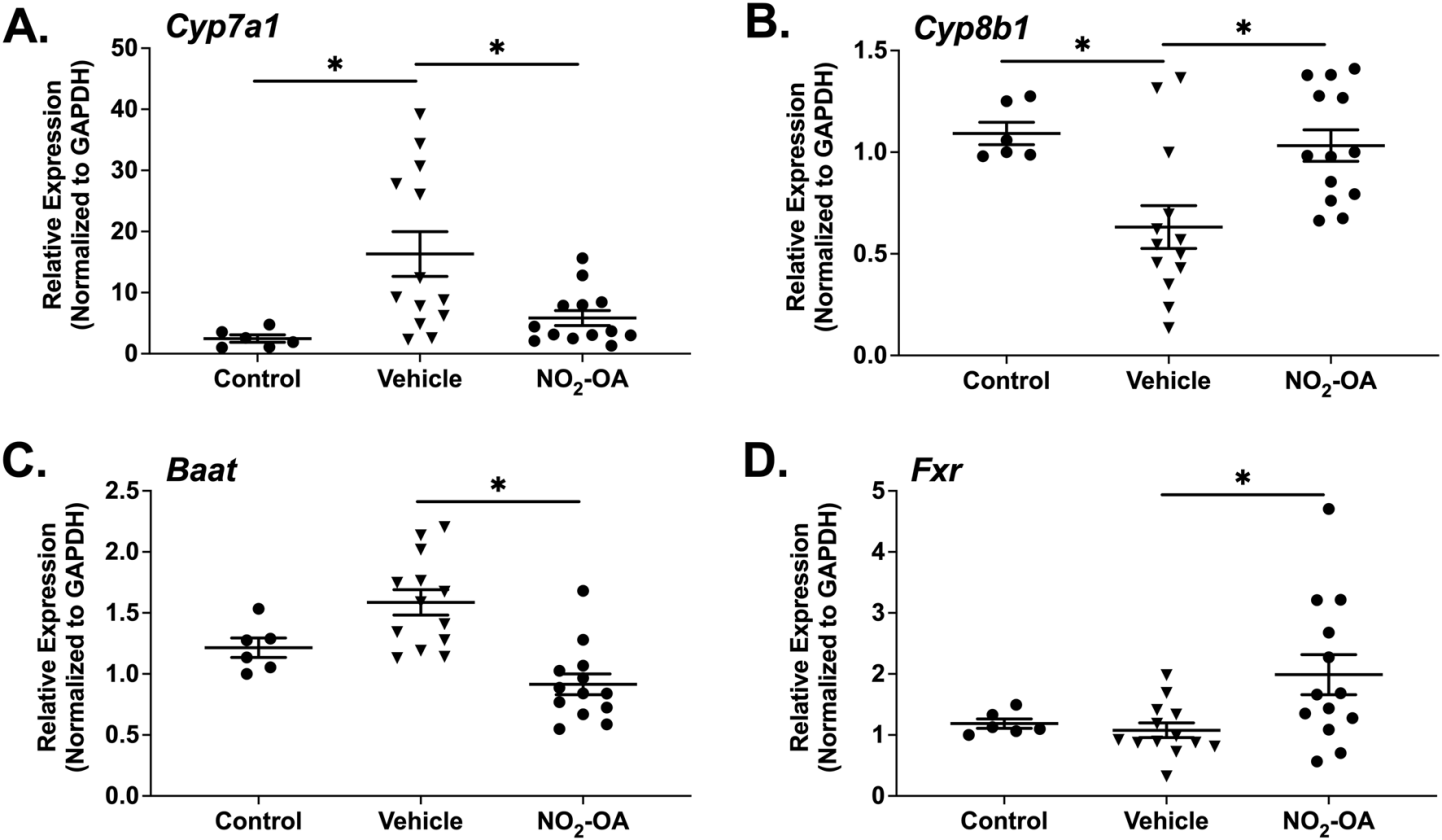
NO_2_-OA treatment abrogates the effects of allergic airway disease on bile acid synthesis. Bile acid synthesis in the liver was evaluated in obese mice with and without AAD following vehicle or NO_2_-OA treatment using real time PCR. *Cyp7a1* **(A)**, *Cyp8b1* **(B)**, bile acid-CoA *N*-amino acyltransferase (*Baat,* **C**), and *Fxr* **(D)** mRNA expression were normalized to *Gapdh* and expression shown is relative to obese control mice, which received mock sensitization and all HDM challenges. n = 6–13 per group, *p < 0.05.

Next, we examined how AAD may affect bile acid synthesis and whether NO_2_-OA impacts these responses by assessing expression of genes necessary for bile acid production and conjugation in the liver. Obese mice with AAD had increased hepatic cytochrome P450 (*Cyp)7a1* mRNA expression and NO_2_-OA treatment in these mice decreased *Cyp7a1* expression to the level detected in obese controls (Fig. 6A). Further, the expression of *Cyp27a1*, which converts cholesterol to 27-hydroxycholesterol, was not altered by AAD or NO_2_-OA treatment compared to obese control mice (Fig. S4A).

AAD also decreased expression of sterol 12α-hydroxylase, *Cyp8b1*, in the livers of obese mice, and NO_2_-OA treatment rescued the suppression of *Cyp8b1*, returning expression levels to those of obese controls (Fig. 6B). The expression levels of *Cyp2c70* were next evaluated to determine whether AAD modulated the conversion of chenodeoxycholic acid to muricholic acid. A modest increase in *Cyp2c70* expression was observed in obese mice with AAD compared to obese controls that was reduced by NO_2_-OA treatment; although, these changes were not statistically significant (Fig. S4B). NO_2_-OA significantly decreased the expression of bile acid-CoA:amino acid *N*-acyltransferase (BAAT), which conjugates bile acids to either glycine or taurine (Fig. 6C). *Baat* expression in the liver was upregulated in obese mice with AAD, although not statistically significant (p = 0.0661). Finally, NO_2_-OA treatment of obese mice with AAD resulted in a significant increase in *Fxr* expression in the liver compared to obese mice with AAD treated with vehicle and obese controls. Thus, NO_2_-OA-mediated induction of FXR may be one mechanism whereby NO_2_-OA regulates bile acid synthesis in the liver (Fig. 6D). In aggregate, these results indicate that NO_2_-OA abrogates AAD-mediated up-regulation of bile acid synthesis and conjugation resulting in decreased levels of βMCA and tβMCA compared to obese mice with AAD.

## Discussion

Obesity-associated asthma is a disease with multiple phenotypes and diverse clinical presentations^21^. Asthma is more prevalent in obese individuals, but not all individuals who are obese present with asthma, despite having a higher risk of developing disease and dysregulated pulmonary function. Herein, the novel discovery that changes in bile acid profiles modulate lung function is reported. This insight can motivate further studies linking specific bile acid metabolites to altered lung function, as we strive to understand and treat disease pathogenesis in complicated disease phenotypes such as obesity-related asthma.

Altered bile acid profiles have been reported in asthmatic patients, with GCA, glycodeoxycholate, taurochenodeoxycholate and taurocholate increased with asthma, compared to healthy individuals^6,15,16^. Primary bile acids are synthesized in the liver by a series of cytochrome P450-mediated reactions^22,23^. The classical pathway accounts for 90% of bile acid synthesis and is initiated by the 7α-hydroxylation of cholesterol by the 7α-hydroxylase, CYP7a1^22^. HDM sensitization and challenge resulted in a significant increase in *Cyp7a1* mRNA expression compared to obese controls. CYP7a1 expression is regulated by FXR as induction of FXR recruits the small heterodimer protein, SHP, to suppress CYP7a1^12^. The observed induction of hepatic *Fxr* expression is one possible mechanism by which this nitroalkene may alter AAD.

Further examination revealed that HDM-induced AAD perturbed the ratio of cholic acid to chenodeoxycholic acid by down-regulating sterol 12α-hydroxylase, *Cyp8b1*, expression. This in turn increases levels of chenodeoxycholic acid, the substrate of CYP2c70 metabolism that yields α-/β-muricholic acids^24^. AAD also increased conjugation of βMCA with the rodent preferred conjugate, taurine, in a reaction catalyzed by hepatic BAAT, resulting in an increase in tβMCA in both serum and lung. NO_2_-OA significantly abrogated the effects of AAD on bile acid synthesis and conjugation to restore mRNA expression levels to those of the obese controls. Weight gain alone may also contribute to the increase in the level of tβMCA observed in this model. Murine models of diet-induced obesity show decreased microbial diversity that results in decreased bile salt hydrolase activity, and consequently an increase in conjugated bile acids^25,26^. Increased conjugated bile acid levels are also detected in distal organs of germ-free rats^27^, but any contributions to specific disease pathogenesis remains undefined.

Notably, bile acids act as agonists or antagonists for their cognate receptors and promote signaling in opposing effects depending on the target tissue and other underlying factors^28–32^. This in part motivated us to define the impact of endogenous bile acid signaling in the lung and specifically in asthma. In individuals with mild-moderate asthma, we discerned that levels of the conjugated bile acids GCA and GUDCA were associated with BMI and asthma status, and that BMI alone may play a predominant role as demonstrated by the strengthened correlation between GUDCA and FEV_1_ when only considering subjects with a BMI > 25^13^. Although strong correlations between serum bile acid levels and FEV_1_ are shown, the cohort assessed is small and it is not possible to determine or control for the effects of race and gender differences, early versus late onset of disease or stratify by Th2 phenotype. While there was no correlation with the Type 2 inflammation biomarker fractional exhaled nitric oxide, GUDCA levels did trend toward a negative correlation with blood eosinophils and the correlation between GUDCA and FEV_1_ was stronger in subjects with a BMI > 25. Thus, it may be that GUDCA levels are more related to weight gain rather than airway hyperreactivity (Fig. S1). Furthermore, systemic changes related to metabolic syndrome and corresponding therapies may also contribute to changes in bile acid metabolism. The effects of metabolic syndrome on our findings were also limited by cohort size. With these limitations in mind, future studies will focus on larger analyses of obese asthma and other asthma endotypes that have been stringently characterized.

In this study, NO_2_-OA reduced systemic and pulmonary levels of βMCA and tβMCA in obese mice with AAD. NO_2_-OA and the pure positional isomer, 10-NO_2_-OA (CXA-10), have been used in cell and murine models of obesity, inflammation, and fibrosis. In human Phase 1 studies the pleiotropic effects of CXA-10 were evaluated in overweight/obese males. CXA-10 (P.O., daily, 2 wk) activated multiple tissue defense mechanisms and inhibited pro-inflammatory signaling responses. These basic research and clinical studies have produced reproducible data that has culminated into a profile of targets in various disease pathologies that include a) the inhibition of NF-kB-regulated inflammatory cytokine, adipokine (leptin and adiponectin) and adhesion molecule expression^20,33,34^, b) the inhibition of pro-inflammatory macrophage activation^35,36^, c) the activation of Nrf2-regulated adaptive gene expression^37,38^ and d) the prevention and reversal of fibrosis^19,36,39,40^. Based on this data, a clinical trial administering CXA-10 for 6 weeks is ongoing in obesity-associated asthma (NCT03762395). The present findings encourage a therapeutic potential for small molecule, electrophilic nitroalkenes in obesity-associated asthma, as it improves lung function, specifically small airway resistance and tissue elastance. The lowering of these viscoelastic parameters of the lung parenchyma and reduction of bile acids following NO_2_-OA treatment suggests a contribution of bile acids to small airway dysfunction. Further, NO_2_-OA/CXA-10 may have unexplored effects on bile acid formation via activation of liver FXR, which warrants future investigation.

In summary, we report the novel linkage between asthma pathogenesis and dysregulated bile acid synthesis/levels in the setting of obesity. A more detailed investigation into the spectrum of bile acids and their pulmonary actions is needed to better understand if changes in bile acid profiles are caused directly by obesity and allergen exposure or indirectly due to obesity-mediated changes of the gut microbiome and gut barrier integrity. These results also extend the mounting evidence that small molecule electrophiles act pleiotropically to regulate metabolism. Bioanalytical studies linked with the ongoing clinical trial of CXA-10 effects on individuals with obesity-associated asthma will lend better perspective as to how electrophilic nitroalkenes modulate metabolic function.

## Methods

### Human subjects, questionnaires, and spirometry

Human studies were approved by the Institutional Review Board of the University of Pittsburgh and the University of Colorado in accordance with The Code of Ethics of the World Medical Association. Subjects were recruited through either the Electrophilic Fatty Acid Derivatives in Asthma study at Pittsburgh (PRO11010186) or the University of Colorado Obesity study of Metabolic Dysregulation and the Airway Epithelium in Asthmatics (16-2522). All subjects, 18 to 65 years of age, provided informed consent (Table 1). For both the Pittsburgh and Colorado studies, male and female subjects were nonsmokers in the last year and had a 10 or less pack-year smoking history. Healthy subjects had normal lung function and no history of chronic respiratory diseases with or without atopy. Asthmatic patients had a 12% or greater bronchodilator response to 4 puffs of albuterol or equivalent or PC20 methacholine (16 mg) if no BD response. Asthmatic patients were mild-moderates with an FEV_1_ of greater than 60% of predicted value and were taking either no controller medications up to low- to moderate-dose inhaled corticosteroids (ICS) with or without a second controller agent (leukotriene modifier or long-acting β-agonist) as clinically indicated. Subjects completed blood draws (CBC with differential collected at Pittsburgh site only), fraction of exhaled nitric oxide (FeNO) measurement, and baseline and postbronchodilator spirometry per ATS guidelines^41,42^. Peripheral eosinophil counts were reported as cells per microliter.

### Murine model of obesity-associated asthma

4-week-old, male C57BL/6 mice were purchased from Jackson Laboratory and fed high fat diet (HFD, 60% fat diet D12492, Research Diets, Inc.) for 12 weeks and water ad libitum. Experiments are approved by the University of Pittsburgh IACUC (20016689) and in accordance with NIH guidelines. This study was carried out in compliance with the ARRIVE guidelines (https://arriveguidelines.org). Obese C57BL/6 (WT) mice were sensitized with 2 µg of HDM (Greer Lot #213051, endotoxin level 32.25 EU/vial) and cholera toxin adjuvant (0.1 µg) via orophargyneal aspiration on Day 0 and 7. The obese control group only received adjuvant during sensitization so that they do not develop AAD. All groups of mice were then challenged daily for five consecutive days (Day 14–18) with 2 µg HDM by oropharyngeal aspiration. Three days after the last challenge (D21), AAD was assessed. To investigate the therapeutic potential of NO_2_-OA, mice were treated with 25 mg/kg NO_2_-OA via gavage (Days 14–18) three hours prior to HDM challenge. To investigate the contribution of diet to murine bile acid levels, AAD was also induced in age-matched normal chow fed WT mice (lean mice) using the treatment scheme described above.

### Lung mechanics measurements

Pulmonary function was assessed by mechanical ventilation of anesthetized (100 mg/kg pentobarbital, i.p.) and tracheotomized mice using a computer-controlled small-animal mechanical ventilator (flexiVent; SCIREQ, Montreal, Quebec, Canada) as previously described^43–45^**.** Briefly, mice were mechanically ventilated at 150 breaths/min with a tidal volume of 10 mL/kg and a positive end expiratory pressure of 3 cmH_2_O (mimicking spontaneous ventilation). First, the quasi-static mechanical properties of the lung at baseline (compliance and hysteresis) were measured using pressure–volume curves (stepwise pressure-driven maneuvers). Secondly, respiratory system mechanics were assessed by alternating perturbations of the single (SnapShot-150) and broadband frequency forced oscillation techniques (Quick Prime-3) prior to (baseline) and following inhalation of increasing doses of aerosolized methacholine (0–50 mg/mL). Multiple linear regression was used to fit measured pressure and volume in each individual mouse to the model of linear motion of the lung^46,47^. Model fits that resulted in a coefficient of determination < 0.9 (constant phase model; multiple frequency forced oscillation perturbations) and < 0.95 (single compartment model, single frequency forced oscillation perturbations) were excluded. Total respiratory resistance (*R*_*rs*_) and elastance (*E*_*rs*_) as well as airway resistance (R_n_, Newtonian resistance), tissue damping (G, tissue resistance) and tissue elastance (H, tissue elastance) contributions were calculated using Flexiware 8.2.0 software (https://www.scireq.com/flexivent/flexiware/, SCIREQ, Montreal, Quebec, Canada). The response to methacholine at each dose was reported as the highest measurement for each parameter of airway mechanics within the first ten perturbations following methacholine aerosolization.

### Bronchoalveolar lavage and tissue harvest, processing, and histology

After measuring respiratory mechanics and sacrifice by pentobarbital, blood was collected via cardiac puncture and processed in tiger top tubes for serum to be used for bile acid measurements and ELISA. Then, BAL fluid was obtained and total and differential cell counts were performed as previously described^44,45^. BAL fluid supernatants were stored at − 80 °C until analyses. Right lung lobes were harvested, and flash frozen for RNA, Bioplex cytokine and chemokine assay (Bio-Rad), and metabolomics analyses. Left lung lobes were fixed with 10% formalin, mounted in paraffin, and 5-μm sections were prepared for histopathology (StageBio, Mt Jackson, VA).

### Histological analyses

5-μm sections of lung tissue were obtained and stained with hematoxylin and eosin or periodic acid Schiff (PAS) stain for assessment of pulmonary inflammation and mucus production as previously described^44,45^. To characterize tissue inflammation in the lung, hematoxylin and eosin-stained sections were scored by a pathologist (T.D.O.) who was blinded to sample groups. Individual fields were examined throughout the entire lung section using a light microscope (100 × magnification). Scoring in each field was based on the percentage of tissue with inflammation according to the following scale: 0 = no inflammation, 1 = up to 25%, 2 = 25%–50%, 3 = 50%–75%, 4 = 75%–100%. The inflammation score was then reported as a ratio of the sum of all of the scores divided by the total number of fields counted for each sample. Next, mucus production was quantified by a pathologist (T.D.O.), who was blinded to the identity of the sample groups. Each bronchiole was examined throughout the entire PAS-stained lung section (100 × magnification) and the number of bronchioles with positive staining was recorded. The PAS score was reported as the ratio of the number of PAS-positive bronchioles divided by the total number of bronchioles counted for each sample.

### Targeted bile acid analysis by SID-LC-HRMS

#### Sample preparation

Serum (50 µL) and lung homogenates were extracted with modifications to published protocols^48,49^. Briefly, internal standards were added to 50 µL of serum and bile acids were extracted with 500 µL of ACN + 3% HCl containing a final internal standard mix (75 ng/mL). Samples were vortexed and spun down at 14,000 × *g* for 15 min. Supernatant was transferred to a different vial and dried for 2 h by speedvac. Samples were reconstituted in 1:1 MeOH:H_2_O (50 µL) for analysis. Lung tissue was processed in the same manner except tissue was homogenized in the solvent system described above at a ratio of 15 µL/mg before centrifugation and drying under vacuum.

#### SID-LC-HRMS

Targeted analysis of bile acids was conducted using a Vanquish UHPLC coupled to either a Q Exactive or ID-X mass spectrometer (Thermo Fisher Scientific, Waltham, MA). Samples (20 µL) were injected onto a Phenomenex Luna C18 column (2 × 100 mm, 5 µ) and separated over a 20 min gradient at a flow of 0.6 mL/min. Solvent A consisted of aqueous 5 mM ammonium acetate with 0.12% formic acid and Solvent B was MeOH. The gradient started at 52% B, which increased to 100% B from 0.4 to 13.5 min and was held for 2 min before returning to baseline conditions. Bile acids were identified by their accurate mass, retention time and stable isotope labeled internal standards. Bile acids are reported as “Relative Amount”, which is the peak area ratio of the analyte to their corresponding deuterated internal standard for plasma and serum and the peak area ratio normalized to tissue weight for the lungs (Table 2).

### RT-PCR

Livers from obese control mice, and obese mice with AAD treated with vehicle or NO_2_-OA were pulverized in liquid nitrogen. Approximately 30 mg was weighed into 1 mL of Trizol (Invitrogen) for RNA extraction per manufacturer’s instructions. Next, cDNA was prepared according to iScript cDNA Synthesis kit (BioRad) according to the manufacturer’s instructions. Real-time PCR was performed with TaqMan Fast Advanced PCR Master mix (Applied Biosystems) and relative gene expression was calculated using established methods^50^.

### Statistics

All analyses were performed using GraphPad Prism 7 (GraphPad Software Inc., La Jolla, CA). Experiments involving one or two variables were analyzed by one-way analysis of variance with Tukey’s post-hoc or with Welch’s test or two-way analysis of variance with a Bonferroni post-hoc test, respectively. Data comparing two groups were analyzed using an unpaired t-test. Data was tested for normality using Shapiro Wilks and natural log transformed when required for analysis by a Pearson χ^2^ test. For demographic categorical and continuous variables, Kruskal–Wallis test and Pearson χ^2^ tests were used, respectively. An overall p < 0.05 was considered statistically significant. Data shown are mean ± SEM.

## Supplementary Information

Supplementary Figures.

## Abbreviations

AAD: Allergic airway disease
BAAT: Bile acid-CoA:amino acid *N*-acyltransferase
BAL: Bronchoalveolar lavage
BMI: Body mass index
Ers: Pulmonary elastance
FEV1: Percent predicted forced expiratory volume
FXR: Farnesoid X receptor
G: Tissue damping
GCA: Glycocholic acid
GUDCA: Glycoursodeoxycholic acid
H: Tissue elastance
HDM: House dust mite
NO_2_-OA: Nitro-oleic acid
Rn: Airway resistance
Rrs: Pulmonary resistance
SID-LC-HRMS: Stable isotope dilution liquid chromatography high resolution mass spectrometry
tβMCA: Tauro-β-muricholic acid
βMCA: β-Muricholic acid
