# Supplementary material for: Nitroalkene fatty acids modulate bile acid metabolism and lung function in obese asthma
Source: Sci Rep. 2021 Sep 7;11:17788. doi: 10.1038/s41598-021-96471-9 (PMC8423735; doi:10.1038/s41598-021-96471-9)
Supplement: Supplementary file 1 — Supplementary Figures. [file 41598_2021_96471_MOESM1_ESM.docx]

**
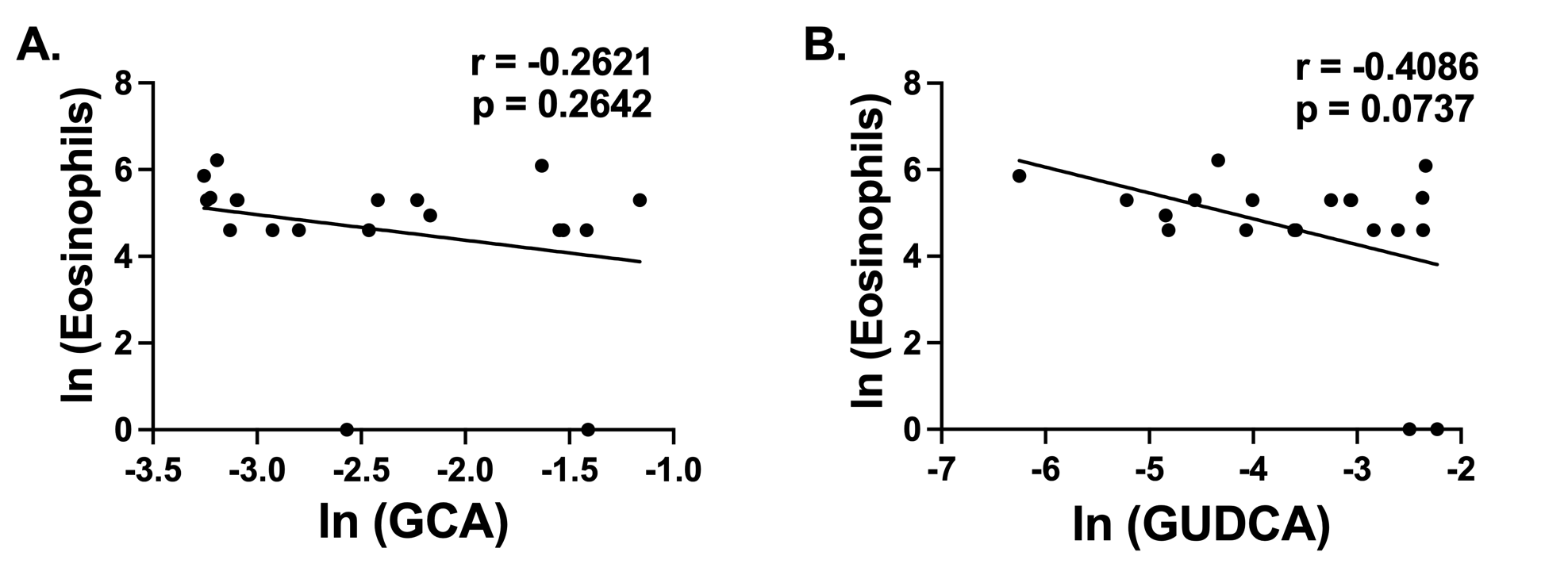
**

**Supplemental Figure 1. GCA and GUDCA do not correlate with absolute serum eosinophil counts of individuals with asthma.** Pearson correlations of serum GCA (A) and GUDCA (B) levels and blood eosinophil counts from patients with asthma. Graphs show natural log transformed data. n=20 pairs

**
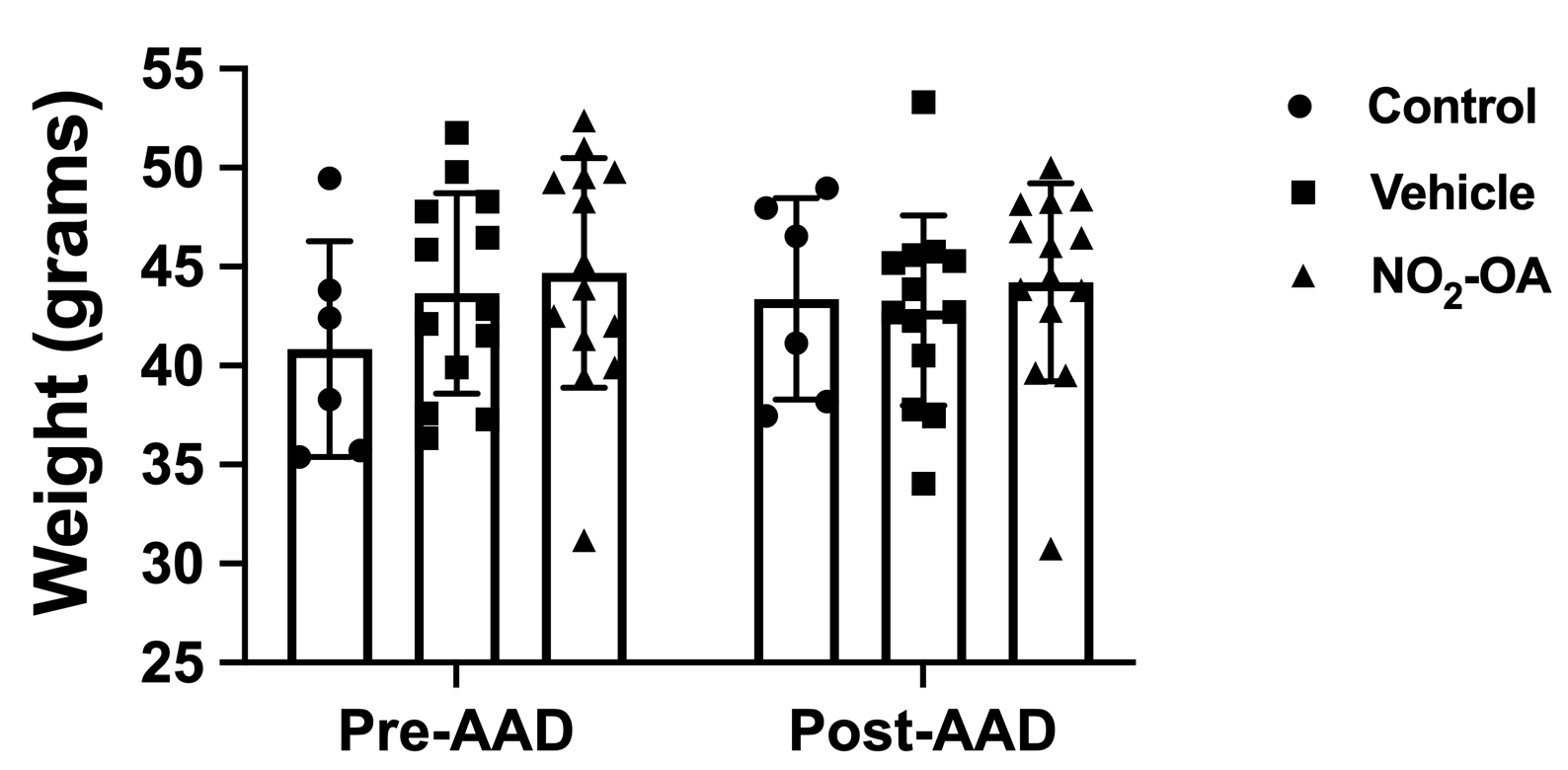
**

**Supplemental Figure 2. Weight of obese mice pre- and post-allergic airway disease (AAD).** Mice fed high fat diet were weighed pre- and post-AAD and treatment with NO_2_-OA (n=14) or vehicle control (n=13). Obese control mice received mock sensitization and all HDM challenges and did not develop AAD (n=6).


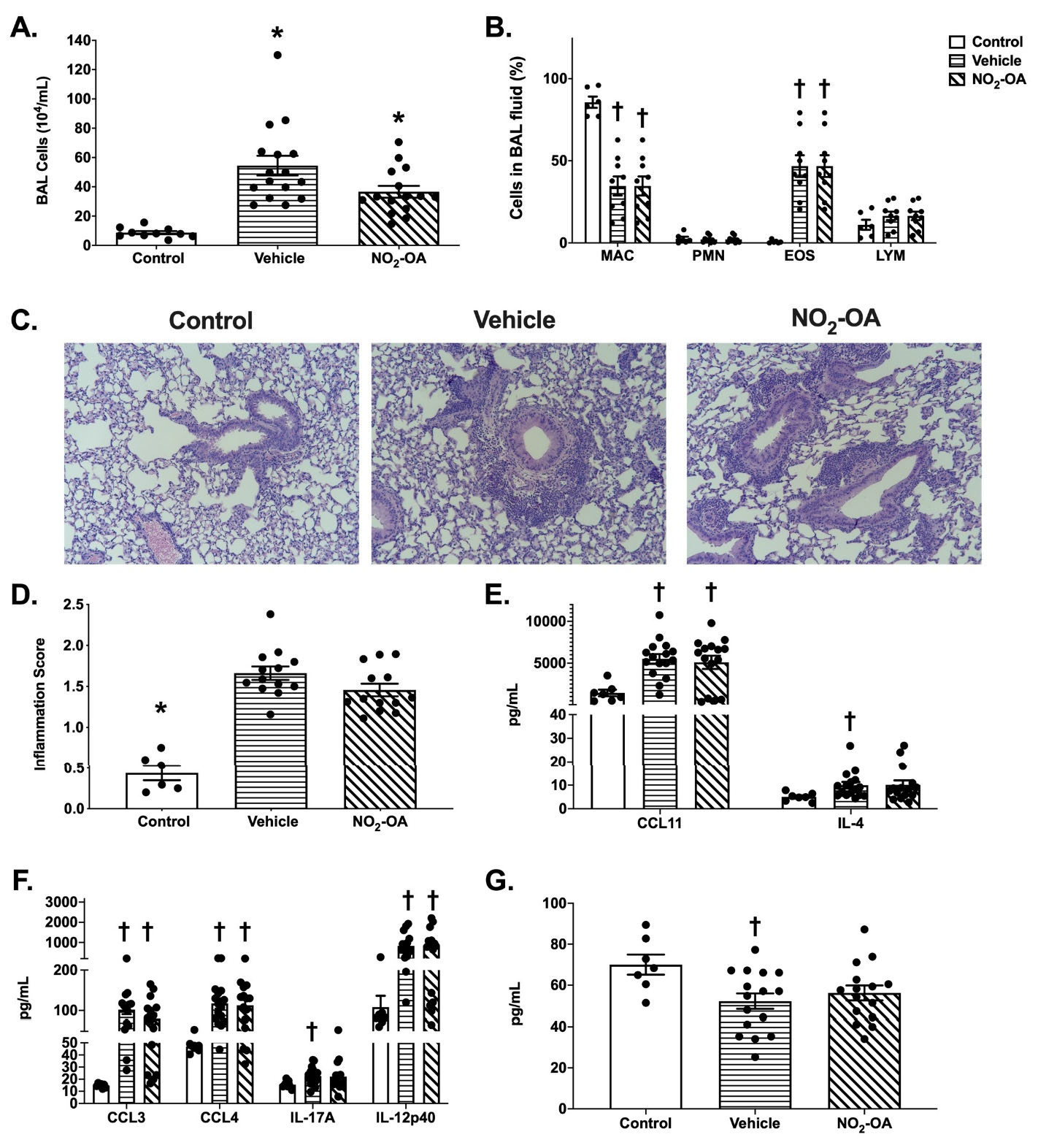


**Supplemental Figure 3. NO_2_-OA treatment does not significantly alter inflammation in the lungs of obese mice with AAD.** Obese C57BL/6J mice were sensitized and challenged with house dust mite (HDM) to induce allergic airway disease (AAD). Mice with AAD were orally gavaged with either triolein (Vehicle) or 25 mg/kg nitro-oleic acid (NO_2_-OA). Cellular inflammation in the airspaces was measured by (A) total cells per mL of BAL fluid and (B) BAL fluid cell differential counts. Obese control mice received mock sensitization and all HDM challenges. (C) Representative images of hematoxylin and eosin-stained lung sections (x100 magnification) were observed and (D) inflammation was quantified by a pathologist. Graphs show data for control (n=6-10), vehicle (n=9-16), and NO_2_-OA (n=10-16) combined from four independent experiments. (E&F) Pro-inflammatory Th2- and Th17-related cytokines and chemokines (IL-4, IL-17, IL-12p40, CCL3, CCL4, and CCL11) and (G) the anti- inflammatory cytokine, IL-10, were measured in the lungs using a Bioplex immunoassay kit. *p<0.05 when compared to all other groups. †p<0.05 when compared to the control group.

**
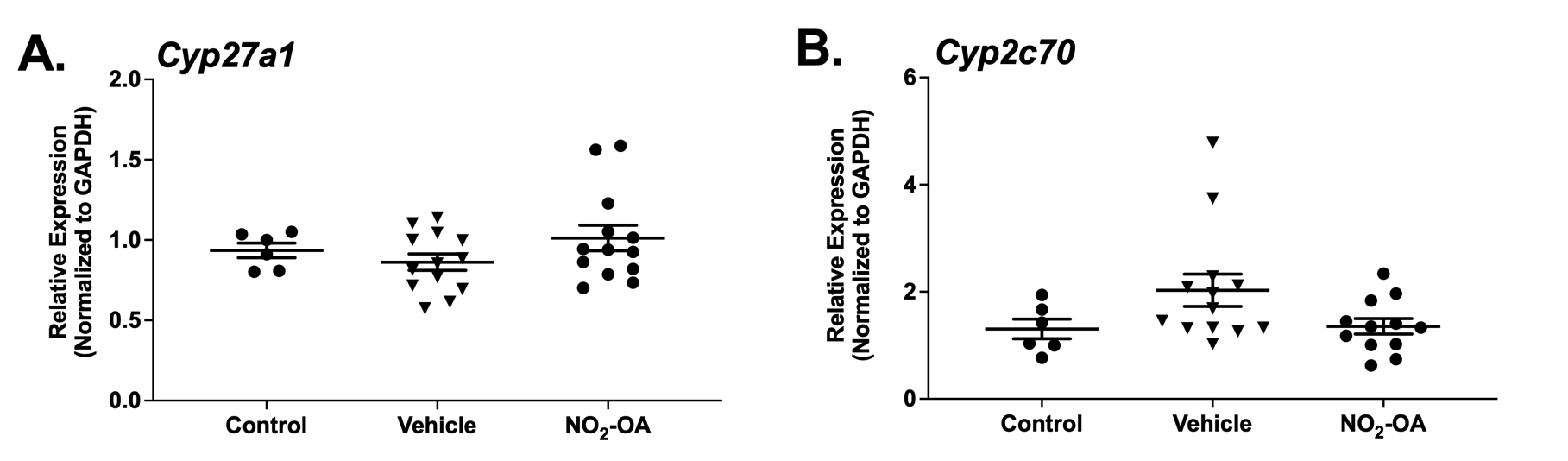
**

**Supplemental Figure 4. *Cyp27a1* and *Cyp2c70* mRNA expression are not altered in obese mice.** *Cyp27a1* (A) and *Cyp2c70* (B) mRNA expression from the livers of obese mice without AAD (control, n=6) and obese mice with AAD following vehicle (n=13) or NO_2_-OA (n=13) treatment was measured by real time PCR. Gene expression shown is normalized to GAPDH and relative to obese control mice.
